# Evaluating a volunteer ‘Health Champions’ intervention supporting people with severe mental illness to manage their physical health: feasibility hybrid randomised controlled trial

**DOI:** 10.1192/bjo.2024.746

**Published:** 2024-10-04

**Authors:** Julie Williams, Ray McGrath, Karen Ang, Ioannis Bakolis, Andy Healey, Jorge Arias de la Torre, Isabel Mdudu, Fiona Gaughran, Euan Sadler, Mariana Pinto da Costa, Errol Green, Natalia Stepan, Gracie Tredget, Zarnie Khadjesari, Sean Cross, Nick Sevdalis

**Affiliations:** Centre for Implementation Science, Health Service and Population Research Department, Institute of Psychiatry, Psychology and Neuroscience, King's College London, UK; South London and Maudsley NHS Foundation Trust, London, UK; and King's Health Partners, London, UK; Care in Long Term Conditions Research Division, King's College London, UK; CIBER of Epidemiology and Public Health (CIBERESP), Centro de Investigación Biomédica en Red, Madrid, Spain; and Institute of Biomedicine (IBIOMED), Universidad de León, Spain; Volunteer Service, South London and Maudsley NHS Foundation Trust, London, UK; National Psychosis Unit, South London and Maudsley NHS Foundation Trust, London, UK; and Department of Psychosis Studies, Institute of Psychiatry, Psychology and Neuroscience, King's College London, UK; Department of Nursing, Midwifery and Health, School of Health Sciences, Faculty of Environmental and Life Sciences, University of Southampton, UK; South London and Maudsley NHS Foundation Trust, London, UK; and King's College London, UK; Quality Centre, South London and Maudsley NHS Foundation Trust, London, UK; Mind and Body Programme, King's Health Partners, London, UK; Behavioural and Implementation Science (BIS) Research Group, School of Health Sciences, University of East Anglia, UK; Centre for Implementation Science, Health Service and Population Research Department, Institute of Psychiatry, Psychology and Neuroscience, King's College London, UK; and Mind and Body Programme, King's Health Partners, London, UK; Centre for Behavioural and Implementation Science Interventions, Yong Loo Lin School of Medicine, National University of Singapore, Singapore

**Keywords:** Psychosocial interventions, Psychotic disorders/schizophrenia, Mental health services, Patients and service users, Community mental health teams

## Abstract

**Background:**

People with severe mental illness (SMI) have worse physical health than the general population. There is evidence that support from volunteers can help the mental health of people with SMI, but little evidence regarding the support they can give for physical health.

**Aims:**

To evaluate the feasibility of an intervention where volunteer ‘Health Champions’ support people with SMI in managing their physical health.

**Method:**

A feasibility hybrid randomised controlled trial conducted in mental health teams with people with SMI. Volunteers delivered the Health Champions intervention. We collected data on the feasibility of delivering the intervention, and clinical and cost-effectiveness. Participants were randomised by a statistician independent of the research team, to either having a Health Champion or treatment as usual. Blinding was not done.

**Results:**

We recruited 48 participants: 27 to the intervention group and 21 to the control group. Data were analysed for 34 participants. No changes were found in clinical effectiveness for either group. Implementation outcomes measures showed high acceptability, feasibility and appropriateness, but with low response rates. No adverse events were identified in either group. Interviews with participants found they identified changes they had made to their physical health. The cost of implementing the intervention was £312 per participant.

**Conclusions:**

The Health Champion intervention was feasible to implement, but the implementation of the study measures was problematic. Participants found the intervention acceptable, feasible and appropriate, and it led them to make changes in their physical health. A larger trial is recommended, with tailored implementation outcome measures.

People diagnosed with a severe mental illness (SMI), such as schizophrenia or bipolar disorder, experience inequalities in their physical health compared with the general population.^1,2^ This includes having multiple long-term conditions and a shorter life expectancy of approximately 10 years.^3^ The underlying causes of this are complex and multifaceted, located at individual, service organisation and societal levels.^4^ Approaches to address these health inequalities at the individual level have included interventions to support people with SMI to lose weight,^5^ be more physically active^6^ and manage specific illnesses (e.g. diabetes), with varying levels of success. At a service organisation level, there is evidence that people with SMI report challenges in navigating complex healthcare services,^7^ and can be affected by ‘diagnostic overshadowing’, whereby healthcare professionals attribute physical health concerns to their mental illness.^8^ At the societal level, stigma and discrimination towards people with SMI can negatively affect their day-to-day living and experiences, including in interactions with healthcare services and professionals.^9^

One promising potential approach that could help individuals with SMI manage their physical health is the use of volunteer support. Volunteers are recognised as providing value in healthcare settings,^10^ and are explicitly mentioned in national policy such the NHS Long Term Plan in England^11^ and the Volunteering Taskforce report.^12^ Volunteer provision of individual support for people with SMI improves mental health, increases social contacts and reduces loneliness and social isolation among those with SMI.^13–16^ This type of volunteering also benefits the volunteers themselves, through feeling useful and acquiring new skills.^16^ To date, we know of no studies that have evaluated whether it is feasible to deliver a volunteer intervention to support the physical health of people with SMI, and the potential health impacts such an intervention may have.

## Aims of the study

This study aimed to evaluate the feasibility and acceptability of an intervention where trained volunteer ‘Health Champions’ support people with SMI in managing their physical health, compared with treatment as usual. We were interested in the feasibility in terms of being able to recruit patients and Health Champions, and whether both groups engaged with the intervention. We did not predefine the numbers needed to progress to a larger trial. We collected data on implementation challenges and clinical and economic metrics, to inform a potential larger-scale trial evaluation.

## Method

### Study design

This was a feasibility hybrid randomised controlled trial (RCT), which evaluated both clinical and implementation outcome measures and analysed costs, but was not powered to assess changes in clinical, implementation or cost-effectiveness outcomes.^17^ Detailed methods and design were reported in the published study protocol.^17^

### Setting

This study took place in community mental health teams (CMHTs) in the South London and Maudsley NHS Foundation Trust (thereafter, the ‘Trust’) in London, UK. The Trust provides secondary mental health services for four London boroughs of Croydon, Lambeth, Lewisham and Southwark.

We collected data from both patients and volunteer Health Champions.

### Patient recruitment

Patients were recruited directly from CMHTs with staff in the teams identifying people who may be eligible; or by using the Trust's Consent for Contact (C4C) service (https://slam.nhs.uk/consent-for-contact/) to identify people who had previously consented to be approached by researchers to take part in research projects. A baseline assessment which included the clinical outcomes (see relevant section below) and questions about why the person wanted to take part in the study was then conducted by telephone. Following this assessment, patients were randomised to either the intervention or control group.

Inclusion criteria for patients were as follows: aged 18 years or above; diagnosis of an SMI, including schizophrenia, bipolar disorder, schizoaffective disorder, delusional and other non-mood psychotic disorders, and major depression; wanting to make changes to their physical health; capacity to give written informed consent to take part in the study in the English language and able to provide a named care coordinator or other point of contact in the CMHT who would be reachable in the event of a health crisis.

### Health Champions recruitment

Health Champions were recruited from existing Trust volunteers in accordance with Trust policies.

Health Champion eligibility criteria were as follows: existing Trust volunteer who had completed Trust volunteer training; aged 18 years or above; Disclosure and Barring Service checked and cleared; able to attend the additional training relevant to the study and willing to commit 1 h per week (average) for up to 9 months, for the duration of the study.

### The intervention

Patients were matched with a volunteer Health Champion by a volunteer coordinator. Matching was based on geographical area and interests. We tried to meet any preferences patients had in terms of age, gender and ethnicity. They were paired for 9 months with an expectation of meeting hourly once a week, either face to face or remotely.

The Health Champion's role was to support the patient with the physical health goals that were important to them. In the first session, the patient was encouraged to let the Health Champion know what these goals were. The support that the Health Champion would provide was then agreed between the pair, and could include discussing issues and challenges that the patient was facing, giving advice and participating in activities together. We did not prescribe what the goals could be, or how the support was given. Both patients and Health Champions were given a journal to complete if they wished to keep track of their progress.

Study recruitment started in September 2020 and ended in May 2021. It was initially designed to take place face to face, but adaptations were made to allow remote meetings in line with COVID-19 restrictions at the time of the study. Thus, initially the pairs met remotely, but were able to meet face to face when physical distancing restrictions in the UK were lifted and if/when both parties were happy to do so.

### Health Champions support

Health Champions were supported by the volunteer coordinator and research team throughout the study; this included initial training on the role, monthly group supervision and individual support as needed.

### Control group

Patients in the control group received treatment as usual from their CMHT regarding the management of their physical health, which could include physical health checks as mandated by NHS England (NHS England 2019) and support from a care coordinator. Patients in the control group received a copy of a workbook on managing physical health, which had been developed in the Trust and includes sections on how people can look after their physical health, and a copy of the journal given to patients in the intervention group (Supplementary Appendix 2 available at https://doi.org/10.1192/bjo.2024.746).

### Randomisation

Randomisation was conducted by a statistician at King's College London, independent of the study and blinded to allocation and control group. The randomisation system randomly sequenced the order of the patients and entered them into the study stratifying by local borough of residence. A random number generator was used to assign patients to the intervention or the control group. The randomised numbers were put into sealed envelopes in lists for each borough. After a baseline assessment had been completed, the researcher opened the next envelope in the list for that borough to reveal which group the patient was assigned to. The research team was not aware of the allocation until they opened this envelope.

### Sample size

As this was a feasibility RCT, a power calculation to calculate sample size was not appropriate.^18^ Considering the study resources and nature of the study design in that this was a feasibility study, we aimed for a sample size of 100 patient participants: 50 in the intervention group and 50 in the control group. This number is in line with recommendations for feasibility studies from Lancaster et al^19^ and Sim and Lewis.^20^

### Data collection

Patients in both groups were asked to complete baseline and follow-up assessments, at the end of the 9-month study duration. Patients were reimbursed £10 for their time at each assessment. At follow-up, patients in the intervention group and Health Champions were also invited to take part in an interview about their experiences.

### Ethical approval

The authors assert that all procedures contributing to this work comply with the ethical standards of the relevant national and institutional committees on human experimentation and with the Helsinki Declaration of 1975, as revised in 2013. All procedures involving human patients were approved by Brent Research Ethics Committee (approval number: 20/LO/0214). The trial was registered with ClinicalTrials.gov, under registration number NCT04124744.

### Clinical effectiveness measures

#### Primary outcome: quality of life

The primary clinical effectiveness outcome of interest was quality of life, measured with the EQ-5D-5L, which measures five domains (mobility, self-care, usual activities, pain and discomfort, and anxiety and depression) by using five-point scales.^20^

#### Secondary clinical outcomes

Secondary clinical outcomes were as follows:

- Self-management, using the ten-item Patient Activation Measure;^21^ raw scores are summed and transformed to 0–100 metric (0, lowest activation level; 100, highest activation level).
- Mental health-related quality of life, using the ten-item Recovering Quality of Life (ReQoL) measure;^22^ scores range from 0 to 40, where 0 indicates poorest quality of life and 40 indicates the highest quality of life.
- Treatment burden, using the ten-item Multimorbidity Treatment Burden Questionnaire,^23^ which generates four categories of treatment burden by grouping scores greater than 0 into tertiles: no burden (score 0), low burden (score <10), medium burden (score 10–22),^10–22^ high burden (>22).
- Loneliness, using the six-item De Jong Gierveld Loneliness Scale,^24^ scored from 0 to 6, with 0 being least lonely and 6 being most lonely.
- Self-reported use of physical health services and physical health screenings.
- Sociodemographic information, including age, gender, ethnicity, educational level, living arrangements, employment status and relationship status at baseline.

### Implementation outcomes

#### Primary outcome: acceptability

This was measured with the validated four-item Acceptability of Intervention Measure (AIM).^25^ Each item is scored on a 1–5 point scale; scores can range from 4 to 20, with higher scores indicating higher perceived acceptability.

#### Secondary implementation outcomes

Feasibility and appropriateness were measured using the validated Feasibility of Interventions Measure (FIM) and the Intervention Appropriateness Measure (IAM).^25^ Both are structured as per the AIM above; scores can range from 4 to 20, with higher scores indicating higher perceived feasibility or appropriateness.

#### Qualitative data collection

We also assessed acceptability, feasibility, appropriateness, fidelity, barriers and facilitators, and unintended consequences qualitatively, by interviewing patients and Health Champions.

### Health economics measures

#### Cost of implementing the intervention

A cost analysis was undertaken to identify the cost of the intervention. Key implementation activities and the time spent by staff on each were identified. Where data was not available assumptions were made regarding the time taken to undertake specific activity (e.g. administrative tasks). Staff time was valued with published unit costs (hourly rates) for staff-specific NHS Agenda for Change 2021 band levels (https://www.pssru.ac.uk/project-pages/unit-costs/unit-costs-of-health-and-social-care-2021/). Researcher time allocated to implementation activities was valued based on costings provided by the university employer. All staff costs include salaries, indirect employment costs and institutional overheads. Costs of equipment were included based on current market rates. These capital expenditures were annualised (assuming equipment lifetime of 3 years and discount rate of 3%). This enables equipment costs to be allocated to the period covered by the project (20 months).

#### Cost of wider health service utilisation

Wider healthcare service utilisation was measured with an adapted version of the patient self-report Adult Service Utilisation Schedule (AD-SUS),^26^ administered by a researcher at baseline and at 9 months post-randomisation for both trial groups. Patients were asked to report retrospectively the number of contacts made with (a) general practitioners, (b) hospital out-patient departments (any reason, mental health or acute setting), (c) accident and emergency department (for any reason) and (d) time spent admitted as an in-patient (for any reason). At baseline and at 9 months, the AD-SUS recorded self-reported contacts for the previous 6-month period. Published data on unit costs of health and social care and NHS Reference costs (https://www.england.nhs.uk/publication/2021-22-national-cost-collection-data-publication/) were used to cost all reported service contacts.

### Data analysis

#### Quantitative analysis

A descriptive analysis of baseline covariates and outcomes (both clinical and implementation measures) was carried out with absolute and relative frequencies (*n* and %) for categorical variables and medians and interquartile ranges for continuous variables, because of the small sample size (and the likely non-normal distributions). A description of the outcomes at 9-month follow-up was also performed. All of these analyses were carried out for all participants and stratified by study group (i.e. Health Champions and control groups).

We assessed changes (before versus after) within each arm of the intervention and control condition on our clinical outcomes (e.g. EQ-5D-5L) with the use of linear regression models, adjusting for baseline total score of EQ-5D-5L. Similar models were fitted for our secondary mental health outcomes.

#### Qualitative data collection and analysis

Semi-structured interviews were conducted with a topic guide developed by the research team, which asked about Health Champion's experiences of delivering the intervention and patients’ experiences of taking part in the intervention, informed by the existing literature on implementation outcomes (see Supplementary Appendix 3). Interviews were conducted by J.W. and R.M. either on Microsoft Teams, by telephone or face to face. All interviews were digitally recorded and transcribed professionally. Interview transcripts were then checked by J.W. and R.M. for any errors, and anonymised before qualitative data analysis.

A thematic analysis approach^27^ was used to analyse and synthesise themes developed from the qualitative data. This involved initially coding interviews into themes, using both inductive and deductive coding to identify responses to specific interview questions covered by the topic guide and other aspects of participants’ experiences. Patient and Health Champion interviews were coded first separately and then compared, to look for similarities and differences in experiences within and between both groups. A coding framework was developed by J.W., R.M. and M.P.d.C. after they had read three initial transcripts. J.W. then coded all transcripts and shared this with M.P.d.C., R.M. and E.S. for further discussion and consensus on the themes. Any new themes were discussed as a group and the coding framework was modified accordingly.

### Cost analysis

Intervention implementation costs are presented descriptively. We report the cost of specific implementation activities and their percentage contribution to overall cost of implementation, total cost of implementation and total cost per Health Champion and per trial participant.

Health service utilisation costs were analysed descriptively. We present mean cost values and s.d. for different categories of healthcare usage and total care utilisation cost by trial arm, for the 6-month reporting period before interview at 9 months. Adjusted and unadjusted differences in mean total healthcare utilisation costs are also presented: adjustments were made for baseline cost and EQ-5D-5L health state utility score differences. The statistical precision of adjusted differences is measured with 95% confidence intervals. Our analysis of healthcare use costs post-randomisation is carried out based on study participants who had complete data on service contacts (a complete-case analysis).

All costs (intervention implementation and healthcare utilisation) are presented in 2020–2021 values. Cost analyses were undertaken in Microsoft Excel, version 2406 for Windows and Stata version 17.0 for Windows.

## Results

### Patients

We recruited 48 patients, of whom 27 were randomised to the intervention group and 21 to the control group. Recruitment took place from August 2020 to September 2021, and follow-up ended in June 2022. The sociodemographic characteristics of the patients at baseline are summarised in Table 1. There were no significant differences in any of the sociodemographic factors between the intervention and control groups.

**Table 1:** Baseline characteristics of the patients, by group and total

	Total		Intervention (*n* = 27, 56.25%)		Control (*n* = 21; 43.75%)	
	*n*	%	*n*	%	*n*	%
Gender						
Female	27	56.25	18	47.62	10	58.33
Male	21	43.75	9	52.38	11	41.67
Ethnicity						
Black	22	45.83	10	37.04	12	57.14
White	18	37.50	12	44.44	6	28.57
Mixed/other	7	14.58	4	14.81	3	14.29
Did not want to say	1	2.08	1	3.70	−	−
Education level						
No qualification	3	6.25	2	7.41	1	4.76
GCSE or equivalent	13	27.08	8	29.63	5	23.81
A level or equivalent	16	33.33	8	29.63	8	38.10
Degree or equivalent	15	31.25	8	29.63	7	33.33
Other qualification	1	2.08	1	3.70	−	−
Living arrangements						
Alone	26	54.17	13	48.15	13	61.90
Spouse or partner	4	8.33	2	7.41	2	9.52
Spouse and children	3	6.25	2	7.41	1	4.76
With children	6	12.50	5	18.52	1	4.76
Other relative	4	8.33	1	3.70	3	14.29
Other not related	2	4.17	2	7.41	−	−
Supported accommodation	3	6.25	2	7.41	1	4.76
Employment status						
Employed	8	16.67	4	19.05	4	16.67
Unemployed	33	68.75	21	57.14	12	68.75
Student	7	14.58	2	23.81	5	14.58
Relationship status						
Single	37	77.08	20	74.07	17	80.95
In a relationship	4	8.33	2	7.41	2	9.52
Married	5	10.42	3	11.11	2	9.52
Divorced	2	4.17	2	7.41	−	−
Median age (interquartile range)	39	18.5	41	18	37	13

**Fig. 1:**
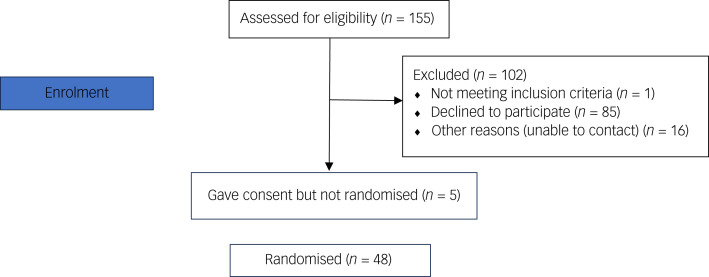
Consolidated Standards of Reporting Trials diagram.

Follow-up data was available for 34 patients, with a total retention rate of 71%, comprising 85% in the intervention group and 52% in the control group (see the Consolidated Standards of Reporting Trials diagram, Fig. 1).

### Clinical effectiveness outcomes

#### Quantitative data

We found no within-group differences in scores (9-month follow-up versus baseline) on the primary outcome (EQ-5D-5L) or any of the secondary clinical outcomes (ReQoL-10; Multimorbidity Treatment Burden Questionnaire; De Jong Gierveld (total), emotional loneliness score, social loneliness score) between the intervention and control groups (Tables 2 and 3).

**Table 2:** Total scores in the primary and secondary outcome measures, overall and by study group

	All patients			Intervention group			Control group		
	*n*	Median	IQR	*n*	Median	IQR	*n*	Median	IQR
Baseline									
EQ-5D-5L	47	0.80	0.36	27	0.76	0.38	20	0.80	0.38
ReQoL-10	48	21.5	12	27	20	11	21	23	15
MMTBQ	48	20	26.25	27	20	35	21	27.5	17.5
De Jong Gierveld Loneliness Scale (total)	47	4	2	26	4	2	21	4	2
Emotional loneliness score	48	2	2	27	2	1	21	2	2
Social loneliness score	47	3	2	26	3	2	21	2	3
Follow-up									
EQ-5D-5L	34	0.78	0.34	23	0.76	0.41	11	0.85	0.39
ReQoL-10	33	24	12	22	23	14	11	28	15
MMTBQ	33	20	22.5	22	25	22.5	11	20	22.5
De Jong Gierveld Loneliness Scale (total)	33	5	3	22	5	3	11	4	3
Emotional loneliness score	33	2	1	22	2	2	11	2	1
Social loneliness score	33	3	1	22	3	1	11	3	2

IQR, interquartile range; ReQoL-10, Recovering Quality of Life, ten-item; MMBTQ, Multimorbidity Burden Treatment Questionnaire.

**Table 3:** Difference between baseline and follow-up primary and secondary clinical outcome scores within the study groups (intra-group), at baseline and follow-up

	Intervention group			Control group		
	*n*	Mean difference	95% CI	*n*	Mean difference	95% CI
EQ-5D-5L	46	−0.01	−0.19 to 0.18	22	0.10	−0.15 to 0.36
ReQoL-10	45	1.68	−3.39 to 6.75	22	2.27	−5.05 to 9.60
MMTBQ	46	0.00	−0.44 to 0.44	22	0	−0.39 to 0.39
De Jong Gierveld Loneliness Scale (total)	44	−0.36	−0.54 to 1.27	22	0.27	−1.30 to 1.85
Emotional loneliness score	44	0.45	−0.13 to 1.04	22	0.36	−0.75 to 1.47
Social loneliness score	45	−0.09	−0.68 to 0.50	22	−0.09	−1.12 to 0.94

Model 1, adjusted for baseline score in the questionnaire; model 2, adjusted for baseline score in the questionnaire and gender, ethnicity, education level, living arrangements, employment status, relationship status and age. ReQoL-10, Recovering Quality of Life, ten-item; MMBTQ, Multimorbidity Burden Treatment Questionnaire.

### Implementation outcomes

#### AIM

Six patients and six Health Champions completed this measure. Both patients and Health Champions had a median score of 17.5 (range 15–20). The interquartile range for both was 4.

#### Secondary outcomes: FIM and IAM

Four patients and three Health Champions completed the FIM, with a median score of 16 (range 15–20). Six patients and six Health Champions completed the IAM, with a median score of 16 (range 16–20).

#### Adoption and sustainability

Twenty-seven patients were randomised to the intervention group. Three were not matched with a Health Champion because they withdrew from the study. Of the 24 matched patients, three had an introduction session only, and 21 had at least three sessions. Fourteen (58%) of the matched patients completed at least 8 months of the intervention; the mean number of months completed was seven. Reasons for people finishing early included changes in patient or Health Champion circumstances or patients feeling that they no longer required the support. The number of sessions received ranged from 3 to 32, with a mean of 20 sessions per participant.

#### Qualitative interviews

Of all patients and Health Champions, 16 patients and 16 Health Champions agreed to be interviewed.

We asked patients about the benefits they had experienced from having a Health Champion. Fourteen participants reported that they had made some changes to their physical health. This included four participants reporting losing weight, and two stating that they were no longer prediabetic. Five patients said that they were doing more exercise, and five had made changes to their diet. One participant had cut down on their tobacco smoking. Two reported that they had developed a more positive attitude to exercise and nutrition. Furthermore, six participants reported that they had made changes to other areas of their life, including being more confident and less anxious.

The main themes from patients’ and Health Champions’ experience of the intervention with illustrative quotes are shown in Table 4. The themes identified are summarised below.

**Table 4:** Qualitative evaluation of implementation outcomes

Implementation outcome	Themes and quotes	
	Patients	Health Champions
Acceptability	Overall, patients found the intervention acceptable. Participants found Health Champions helpful in providing support and encouragement. ‘It's helpful to have the support, someone there to talk to. Yeah, it's good to have someone there to talk to and help meet targets and goals.’ ID2 ‘they give good advice, they give practical things that you can do. The one I had was very cheerful, makes you feel good’ ID10	Health Champions generally found the experience acceptable and enjoyable. ‘It was fun. It was nice talking to him.’ ID11 ‘Overall, I found it very easy and not taxing at all or difficult to navigate.’ HC10
Feasibility	Most patients were happy with how often they met with their Health Champion, and reported that they found taking part easy and that there was good communication with their Health Champion. ‘I really enjoyed it because I though the pair that I was given, the Health Champion I was given was a really good match.’ ID45 ‘we'd just talk on the phone and arrange to meet up like that.’ ID39 ‘I was able to talk about my physical health and what I need to do, what steps I need to take.’ ID42	Most Health Champions found it was feasible to be a Health Champion. For some, if their circumstances changed it became more difficult to continue giving the time needed. ‘I was lucky that I work quite near where she was, so I could go there quite easily.’ ID1 ‘Only lately it's become more complicated because I've started a new job.’ HC4
Appropriateness	Most patients felt that the support from their Health Champion was appropriate for them, it was relevant and suited their needs. ‘It's not a push thing. So, that's what's made it easier for me. That's probably why I lost the weight and probably why I feel like I could carry it on. It didn't feel like no burden or no pressure on me.’ ID22	Most Health Champions found the role appropriate for them and their life. ‘I volunteered cos I knew by doing this it's gonna empower me to get back out and to be able to connect. And it 100% has done that.’ HC6 ‘So, that was alright, even though it is a difficult thing, but it never felt overwhelming.’ HC10
Fidelity	The focus of the intervention was on physical health, but most patients also discussed other aspects of life, as people felt it was not helpful to separate physical health from other aspects of life as they were so inter-related. ‘I have memory loss from the medication, and then she helped me to find a memory game so I can play on something like that.’ ID42 We talked about ‘loads of things: a boyfriend, family, childhood. Lots of conversations.’ ID35	Health Champions also said that separating physical health from other aspects of people's life was often not helpful. ‘If you wanted him to look at his physical health … you had to take into account how much he could do and how much to support him … This way he was supporting his mental health and his physical health.’ ID3 ‘She did talk about her physical health sometimes, But It was usually a much broader conversation.’ ID16
Barriers	Three barriers were identified. One was meeting with their Health Champion if both were busy or if it was done by telephone. ‘it was hard to meet up when I started working extremely hard and my health champion also was working very hard’ ID45 ‘once a week talking to someone over the phone for one hour, you don't get to bond with that person’ ID11 The second was the patients being motivated to make the changes they wanted to make even with the support of the Health Champion. ‘I think it's motivation to actually do the things that I know are good for me … I don't know how to explain it, but I just can't do some things.’ ID35 The third barrier was the impact of COVID-19. ‘if it wouldn't have been for the Covid times, it would've been a lot better. I think it would've been a lot more easier to meet up.’ ID11	The barriers identified included: Defining the role ‘the role was so broad, maybe I tried to do too much or the wrong thing.’ HC1 Communication ‘The biggest challenge was scheduling the calls and actually getting to have the phone calls.’ HC13 Finding time ‘I think the timing wise was just wrong: me trying to do it during the most stressful academic year.’ HC2
Facilitators	The main facilitator identified was the relationship built with the Health Champion-this relationship helped patients to feel listened to and so be able to confide in the Health Champion. ‘She's an amazing person. I really appreciate her time and her empathy’ ID45 ‘‘it's a good match and I also think it's because she was also very knowledgeable when it came to diet and physical exercise. But I also think she was very understanding.’ ID41	Facilitators for Health Champions were: The reward they found doing the role ‘I personally found that really interesting and really rewarding,’ HC7 The supervision sessions ‘It was good because it was a chance for me to hear from other health champions and see how they getting on.’ HC17 ‘I like the way the supervision was because he was saying, ‘If you can make it, it's here for you’.’ HC6 The support from the volunteer coordinator and research team ‘I knew that, when I did start to think, ‘This Is getting a bit too much,’ I felt like I could approach everyone.’ HC2 ‘There was always the opportunity to contact somebody if you needed to … You didn't feel alone at all.’ HC8
Unintended consequences	No unintended consequences were reported. When asked this question, patients reported on life events and the impacts these had had on their experience of the intervention.	
Experience of the intervention	When asked about their experience of the intervention, patients talked about how taking part in the intervention had helped them. ‘It made me feel as though I'd achieved something. So, it helped with my mood. So, it was a goal that I could achieve.’ ID13 ‘I'm happy because I've made a lot of changes to my lifestyle.’ ID35 ‘Before I was having help from [NAME] … I was just in such a dark place and I didn't think I was ever gonna get out of it and get better. But, since the help that I've had, I've been doing a lot every single day.’ ID41 ‘It has made me feel more positive about my physical health and about my self-image.’ ID45	Health Champions talked about what they had got from taking part. ‘Challenging, rewarding and astonishing.’ ID3 ‘It was a good experience because I learnt a lot.’ ID5 ‘The person I was paired with, she was just fantastic.’ ID17

Patients and Health Champions generally found the intervention acceptable, which echoes the findings from the implementation outcome measures. They mostly considered it feasible and appropriate to undertake. In terms of fidelity, patients and Health Champions considered having a focus on physical health alone, which did not consider mental health and other aspects of their life, unhelpful in thinking about making changes because they did not experience these aspects of their life as separate from each other.

Implementation barriers identified by both patients and Health Champions included the social distancing required as a result of the COVID-19 pandemic, and issues with arranging to meet, caused by other considerations such as starting college or long work hours for some Health Champions. For patients, one specific identified barrier was that even with support from a Health Champion, they still found motivation difficult, whereas for a minority of Health Champions, a barrier was a perceived lack of clarity about their role, as this made them unsure of their role.

The main implementation facilitator for patients was the relationship built with the Health Champions, with trust commonly cited as a key factor enabling them to be open about their experiences, which helped them to make changes to their physical health. For Health Champions, two main implementation facilitators were the reward they enjoyed from the role, and the support they received both in supervision sessions and from the project team. This support helped them to feel secure in the role.

No unintended consequences were reported from patients taking part in the intervention or Health Champions delivering it. Patients also reported on their experience of the intervention, in particular noting that having a Health Champion was seen as a powerful factor that allowed them to make changes to their life, such as making changes to their physical health.

### Cost analysis

Over the period of trial, intervention costs amounted to an estimated total of £8422: £312 per participant and £337 per Health Champion in the intervention arm of the trial. Practitioner time allocated to supervision and support of Health Champion volunteers accounted for the highest proportion of total implementation cost (51%) (Table 5). Further details of costs can be found in Supplementary Appendix 4.

**Table 5:** Costs of the Health Champions intervention

	Cost	Percentage contribution to total intervention costs
Supervision and support of volunteers to be Health Champions	£4279	51%
Training	£2355	28%
Recruitment of Health Champions	£761	9%
Equipment expenditure	£635	8%
Matching	£392	5%
Total	£8422	Not applicable
Cost per Health Champion	£337	Not applicable
Cost per trial participant	£312	Not applicable

Twenty-seven randomised patients completed a health service use questionnaire at 9 months post-randomisation (response rate 56%). For the subsample with complete data, costs arising from wider reported contact with health services were mainly associated with primary care usage, along with out-patient and emergency department visits (Table 6). Unadjusted mean total costs (Table 6) were lower for the intervention group than the control group with complete data (−£606; 95% CI −£1170 to −£42). This difference did not persist after adjusting for baseline covariates (−£345; 95% CI −£909 to £219).

**Table 6:** Healthcare utilisation and total costs over the 9-month Health Champions intervention period

	Control group		
	Mean reported contacts (s.d.)	Mean cost (s.d.)	*n*
General practitioner contacts	7.00 (7.96)	£280 (£319)	11
Out-patient visits	3.36 (2.87)	£673 (£575)	11
Emergency department contacts	0.82 (2.40)	£243 (£713)	11
Acute care bed days	0.09 (0.30)	£86 (£286)	11
Psychiatric bed days	0.00 (0.00)	£0 (£0)	11
Total cost, mean (s.d.)	£1283 (£861)		11
	Intervention group		
	Mean reported contacts/bed days (s.d.)	Mean cost (s.d.)	*n*
General practitioner contacts	4.47 (7.05)	£179 (£283)	17
Out-patient visits	2.06 (2.57)	£413 (£514)	16
Emergency department contacts	0.50 (1.03)	£149 (£307)	16
Acute care bed days	0.00 (0.00)	£0 (£0)	16
Psychiatric bed days	0.00 (0.00)	£0 (£0)	16
Total cost, mean (s.d.)	£938 (£567)		16
	Difference in group mean (*n* = 27)	95% CI	
Mean overall cost of wider healthcare utilisation: unadjusted	£606	−£1170 to −£42	
Mean overall cost of wider healthcare utilisation: adjusted	−£345	−£909 to £219	

Health use reported for a 6-month period before interview at 9 months after the beginning of intervention.

## Discussion

We found that the majority of patients who took part in the Health Champions intervention viewed the experience as acceptable, feasible and appropriate. A total of 63% of patients reported that they had made changes to their physical health, including losing weight and being more physically active. Some reported making other changes such as increasing their confidence. The volunteers also overwhelmingly experienced their involvement as positive.

As this was a feasibility study, it was not powered to detect any differences in the size of the effect between our intervention and control groups, but we were interested in understanding the impact of the intervention. Our analysis was an intention-to-treat analysis and we also conducted an analysis with those participants who had a complete baseline and follow-up data, and we did not observe any changes with the total scores (see Supplementary Appendix 5).

We did not conduct a full economic evaluation. We found some changes in wider healthcare utilisation, with lower costs for the intervention participants. The lower cost of healthcare contacts could indicate that support from Health Champions changed healthcare use; however, there needs to be caution in interpreting any economic findings because of the small sample size, but this is something that could be investigated further in a larger trial.

The perceived benefits reported by the patients are consistent with previous studies exploring volunteer support of people with SMI, which identified that the relationship built between the volunteer and patient was paramount.^28,29^ In our study, patients reported the relationship with their Health Champion as key to making the desired changes to their physical health. Having someone involved who was seen as ‘independent’ of health services was important for some patients, and this has also been reported in other studies.^30^ The main reported barriers for the Health Champions were practical issues, such as their availability changing so they could not give the time they wished to the role. This reflects findings from other studies that this type of volunteering is a significant commitment for volunteers.^31^ We have learned that volunteers appreciate having both group and individual support and a named contact when supporting people with SMI in the community. Making sure volunteers are effectively supported is key to any intervention involving volunteers,^16^ and this support needs to be factored in when costing interventions such as this.^32^

A main strength of this study was that implementation science methodologies were used to rigorously evaluate the feasibility of implementing a novel intervention, including evaluating clinical, implementation and cost-effectiveness in one hybrid trial. There were two main limitations of the study caused by conducting the trial during restrictions due to the COVID-19 pandemic. First, we were not able to recruit the number of patients anticipated, as the recruitment processes that we had planned were interrupted; namely, visiting recruitment sites in person and spending time in the sites to make staff and patients aware of the study. Research has shown that this contact facilitates recruitment.^33^ This barrier may have been temporary and may not be a hindrance in a future evaluation of this intervention. Second, the trial was delayed because of the pandemic, so we were no longer able to undertake assessments 6 months after the intervention finished as originally planned. This meant that we had two assessment periods only (baseline and at end of intervention) instead of the planned three.

This Health Champions intervention has been found to be a feasible and acceptable intervention to support people with SMI with their physical health, with qualitative evidence of perceived benefit. Any organisation that wishes to use this model needs to plan the implementation and evaluation of this approach carefully. Three main aspects need to be considered.

First, in terms of cost, the intervention is relatively low cost, but adequate implementation support for the Health Champions is needed for the intervention to be feasible. This support includes regular supervisory contact with Health Champions to allow them to share and discuss any issues arising, and support if the person they are matched with has any crises.

Second, care needs to be taken in identifying which clinical outcomes are most appropriate and meaningful, and how they should be measured. We found that identifying the best outcome measures to assess clinical effectiveness was challenging. This was not a trial of one physical health condition, so we could not use diagnosis-specific outcomes. Although the trial design was not powered to detect a quantitative difference, it is also possible that the five domains of quality of life measured in the EQ-5D-5L questionnaire were not affected by the intervention. For example, unless a participant had problems with mobility, then having a Health Champion was unlikely to make any changes to this. Two possible solutions should be considered in a larger trial. One solution is that, any assessment of change of each patient's stated goal could be done using an individual. There are methodologies that have been developed to do this, including PSYCHLOPS,^25^ where patients identify and score the areas that are problematic for them, and the difference in their score of these problems before and after an intervention is calculated. This has the benefit of ensuring that any change is related directly to a person's individual daily life. Another solution is the use of consulting advisory groups that include people with lived experience and volunteers in discussions on what outcomes should be measured, which would help ensure that the outcomes chosen were meaningful.^15^

Third, from an implementation perspective, we selected a number of questionnaire scales (i.e. AIM, IAM and FIM) that have been previously evaluated psychometrically and are also brief, and so are expected to be easier to administer.^25^ However, we found that the uptake of the scales was very poor. From our experience in the trial, we suggest that this was sometimes a result of participant fatigue, as they were administered after completing an interview and the other outcome measures. Further, some patients and Health Champions found the wording of the questionnaires difficult, as they did not correspond to their experience of having or being a Health Champion. Consulting potential participants in selecting and potentially editing suitable implementation assessment instruments in the context of a larger study is recommended.

A future definitive trial would be beneficial to understand the mechanisms involved in helping participants to make the behaviour changes necessary to improve physical outcomes, as well as quantifying clinical and cost-effectiveness.

## Supporting information

Williams et al. supplementary material 1 Williams et al. supplementary material

Williams et al. supplementary material 2 Williams et al. supplementary material

Williams et al. supplementary material 3 Williams et al. supplementary material

Williams et al. supplementary material 4 Williams et al. supplementary material

Williams et al. supplementary material 5 Williams et al. supplementary material

## Data Availability

The data that support the findings of this study are available from the corresponding author, J.W., upon reasonable request.
