# Supplementary material for: Evaluating a volunteer ‘Health Champions’ intervention supporting people with severe mental illness to manage their physical health: feasibility hybrid randomised controlled trial
Source: BJPsych Open. 2024 Oct 4;10(5):e172. doi: 10.1192/bjo.2024.746 (PMC11536213; doi:10.1192/bjo.2024.746)
Supplement: Williams et al. supplementary material 1 — Williams et al. supplementary material [file S2056472424007464sup001.docx]

**Appendix 5**

**Baseline characteristics of the study participants with a complete follow up by study group.**

|  | **Total**  **(n: 34)** | | **Intervention group**  **(n: 23)** | | **Control group**  **(n: 11)** | |
| --- | --- | --- | --- | --- | --- | --- |
|  | **n** | **n** | **n** | **%** | **n** | **%** |
| **Gender** |  |  |  |  |  |  |
| Female | 19 | 55.88 | 16 | 69.57 | 3 | 27.27 |
| Male | 15 | 44.12 | 7 | 30.43 | 8 | 72.73 |
| **Ethnicity** |  |  |  |  |  |  |
| Black | 12 | 35.29 | 8 | 34.78 | 4 | 36.36 |
| White | 16 | 47.06 | 11 | 47.83 | 5 | 45.45 |
| Mixed/other | 5 | 14.71 | 3 | 13.04 | 2 | 18.18 |
| Did not want to say | 1 | 2.94 | 1 | 4.35 | - | - |
| **Education level** |  |  |  |  |  |  |
| No qualification | 3 | 8.82 | 2 | 8.70 | 1 | 9.09 |
| GCSE or equivalent | 8 | 23.53 | 7 | 30.43 | 1 | 9.09 |
| A level or equivalent | 10 | 29.41 | 5 | 21.74 | 5 | 45.45 |
| Degree or equivalent | 12 | 35.29 | 8 | 34.78 | 4 | 36.36 |
| Other qualification | 1 | 2.94 | 1 | 4.35 | - | - |
| **Living arrangements** |  |  |  |  |  |  |
| Alone | 16 | 47.06 | 10 | 43.48 | 6 | 54.55 |
| Spouse or Partner | 4 | 11.76 | 2 | 8.70 | 2 | 18.18 |
| Spouse and children | 3 | 8.82 | 2 | 8.70 | 1 | 9.09 |
| With children | 5 | 14.71 | 5 | 21.74 | - | - |
| Other relative | 2 | 5.88 | 1 | 4.35 | 1 | 9.09 |
| Other not related | 2 | 5.88 | 2 | 8.70 | - | - |
| Supported accommodation | 2 | 5.88 | 1 | 4.35 | 1 | 9.09 |
| **Employment status** |  |  |  |  |  |  |
| Employed | 5 | 14.71 | 3 | 13.04 | 2 | 18.18 |
| Unemployed | 26 | 76.47 | 18 | 78.26 | 8 | 72.73 |
| Education | 3 | 8.82 | 2 | 8.70 | 1 | 9.09 |
| **Relationship status** |  |  |  |  |  |  |
| Single | 23 | 67.65 | 16 | 69.57 | 7 | 63.64 |
| In a relationship | 4 | 11.76 | 2 | 8.70 | 2 | 18.18 |
| Married | 5 | 14.71 | 3 | 13.04 | 2 | 18.18 |
| Divorced | 2 | 5.88 | 2 | 8.70 | - | - |
| **Median age (IQR)** | 34 | 41(18) | 23 | 41(20) | 11 | 39(17) |

n: number of individuals; %: percentage; IQR: interquartile range.

**Appendix 5 Total scores in participants with a complete follow up the primary and secondary outcome measures overall and by study group**

|  | **All participants** | | | **Intervention group** | | | **Control group** | | |
| --- | --- | --- | --- | --- | --- | --- | --- | --- | --- |
|  | **n** | **Median** | **IQR** | **n** | **Median** | **IQR** | **n** | **Median** | **IQR** |
| **Baseline** |  |  |  |  |  |  |  |  |  |
| **EQ-5D-5L** | 34 | 0.78 | 0.46 | 23 | 0.75 | 0.48 | 11 | 0.81 | 0.58 |
| **ReQoL-10** | 34 | 20 | 10 | 23 | 20 | 11 | 11 | 23 | 7 |
| **MMTBQ** | 34 | 22.5 | 25 | 23 | 20 | 32.5 | 11 | 32.5 | 15 |
| **De Jong Gierveld scale (total)** | 33 | 4 | 2 | 22 | 4 | 2 | 11 | 4 | 2 |
| Emotional loneliness score | 34 | 2 | 2 | 23 | 2 | 2 | 11 | 3 | 2 |
| Social loneliness score | 33 | 3 | 2 | 22 | 3 | 2 | 11 | 2 | 3 |
| **PAM score** | 34 | 52.9 | 23.8 | 23 | 52.9 | 23.8 | 11 | 52.9 | 14.2 |
| **Follow up** |  |  |  |  |  |  |  |  |  |
| **EQ-5D-5L** | 34 | 0.78 | 0.34 | 23 | 0.76 | 0.41 | 11 | 0.85 | 0.39 |
| **ReQoL-10** | 33 | 24 | 12 | 22 | 23 | 14 | 11 | 28 | 15 |
| **MMTBQ** | 33 | 20 | 22.5 | 22 | 25 | 22.5 | 11 | 20 | 22.5 |
| **De Jong Gierveld scale (total)** | 33 | 5 | 3 | 22 | 5 | 3 | 11 | 4 | 3 |
| Emotional loneliness score | 33 | 2 | 1 | 22 | 2 | 2 | 11 | 2 | 1 |
| Social loneliness score | 33 | 3 | 1 | 22 | 3 | 1 | 11 | 3 | 2 |
| **PAM score** | 34 | 59.3 | 18.4 | 23 | 56 | 12.6 | 11 | 62.6 | 23.8 |

IQR: Interquartile range; EQ-5D-5L: Euro QoL 5D-5L version; ReQoL-10: Recovering Quality of Life – 10; MMBTQ: Multimorbidity Burden Treatment Questionnaire. PAM score: Patient Activation Measure score; p: p value for the difference between intervention and control groups using Mann-Witney U test for age
