# Supplementary material for: Evaluating a volunteer ‘Health Champions’ intervention supporting people with severe mental illness to manage their physical health: feasibility hybrid randomised controlled trial
Source: BJPsych Open. 2024 Oct 4;10(5):e172. doi: 10.1192/bjo.2024.746 (PMC11536213; doi:10.1192/bjo.2024.746)
Supplement: Williams et al. supplementary material 2 — Williams et al. supplementary material [file S2056472424007464sup002.docx]

**Cost of Health Champion intervention (supplementary appendix 5)**

| **Activity** | **Staff type undertaking activity** | **Frequency of activity** | **Hours per unit of activity** | **Total hours** | **Activity cost** | **Comments and assumptions** |
| --- | --- | --- | --- | --- | --- | --- |
| **Recruitment** |  |  |  |  |  |  |
| *Introduction of Health Champions role to all those recruited* | Project research lead (University grade 6) | 35 one-to-one meetings (online) | 0.16 | 2.5 | £138 | In total 35 volunteers were recruited to be a Health Champion. Only 25 became active in the programme. Based on recall of discussions these were assumed to last for an average of 10 minutes. |
| *Volunteer training for new provider volunteers* | Volunteer manager (NHS Band 7) | 1 training session (online) | 1.5 | 1.5 | £99 | One compulsory online training session was undertaken with one NHS practitioner and one unpaid volunteer present (time uncosted). |
| *Administrative tasks* | Volunteer manager (NHS Band 7) | 25 sets of items to process | 0.16 | 4 | £264 | Time allocated to processing Disclosure and Barring Service (DBS) checks and occupational health forms – assumed to be 10 minutes each on average (we have no data on this). We have assumed that 28% of those recruited to be a HC were new NHS provider volunteers based on the % of those who remained involved who were known to be new. |
| **Training in the Health Champions approach** |  |  |  |  |  |  |
| *Set up and planning sessions* | Senior/lead nurse (NHS Band 7) | 1 block of set-up and planning | 6 | 6 | £396 | Based on practitioner recall - 6 hours of time to prepare training material and plan sessions involving two practitioners plus 2 hours of preparation time for organisation consultant. |
|  | Volunteer coordinator (NHS Band 5) | 1 block of set-up and planning | 6 | 6 | £264 |  |
|  | Organisation consultant - coaching expert (NHS Band 8a). | 1 block of set-up and planning | 2 | 2 | £ 150 |  |
| *Introductory online training sessions* | Senior/lead nurse (NHS Band 7) | 8 group on-line sessions | 1.5 | 8 | £528 | 8 compulsory group sessions led by two NHS practitioners introducing the Health Champions approach. These took place between October 2020 and February 2021. Group size varied – maximum of 6 trainees, minimum of 1. Costing is based on how long each session was scheduled to last (1.5 hours), but in practice length of sessions varied. Initially all training was planned to be face-to-face “on-site” which would have carried additional costs due to room hire and refreshments. However, due to the COVID pandemic all sessions were conducted online and therefore additional expenditures that may otherwise be incurred have not been included. |
|  | Volunteer coordinator (NHS Band 5) | 8 group on-line sessions | 1.5 | 8 | £352 |  |
| *Online training in coaching techniques* | Organisation consultant - coaching expert (NHS Band 8a). | 3 group on-line sessions | 1 | 3 | £225 | A single additional session was undertaken led by an organisation consultant with expertise in coaching methods (session not compulsory for Health Champions). Not all trainees attended the extra training that was offered in coaching skills due to logistic difficulties in arranging the additional sessions. If more sessions had been arranged this would have increased the costs of Health Champions training activity. Sessions were scheduled to last for an hour. |
| **Supervision and support provided to Health Champions** |  |  |  |  |  |  |
| *Online group supervision* | Senior/lead nurse (NHS Band 7) | 26 on-line group sessions over 13 months | 1 | 26 | £1,716 | Online group supervisory sessions led by two NHS two practitioners carried out twice per month from October 2020 to Nov 2021. However, due to dwindling numbers of attendees these were substituted with one-to-one supervisory sessions. |
|  | Volunteer coordinator (NHS Band 5) | 26 on-line group sessions over 13 months | 1 | 26 | £1,144 |  |
| *One-to-one online supervisory sessions* | Senior/lead nurse (NHS Band 7) | 5 one-to-one online sessions over remainder of project period. | 0.25 | 1.25 | £83 | See above. One-to-one sessions with two NHS practitioners present were shorter than group sessions – we have assumed 15 minutes based on project lead recall of average session length. |
|  | Volunteer coordinator (NHS Band 5) | 5 one-to-one online sessions over remainder of project period. | 0.25 | 1.25 | £55 |  |
| *Additional online ad-hoc support* | Senior/lead nurse (NHS Band 7) | 47 support on-line contacts | 0.25 | 11.65 | £769 | Recorded information on ad-hoc support for the period October 2021 to December 2021 suggests an average of 2.33 ad-hoc contacts per month involving two NHS practitioners. We have assumed this frequency of contact for the entire period of the study (October 2020 to June 2022), and 15 minutes per contact based on recall of average session length. |
|  | Volunteer coordinator (NHS Band 5) | 47 support on-line contacts | 0.25 | 11.65 | £513 |  |
| **Matching study participants with Health Champions** |  |  |  |  |  |  |
| *3-way introductory phone calls* | Volunteer coordinator (NHS Band 5) | 27 matching meetings | 0.33 | 8.91 | £392 | Most of these introductory meetings between Health Champion and study participants (facilitated by the volunteer coordinator) were carried out over the phone with some face- to-face meetings at the tail end of the project. We have made the simplifying assumption that all were carried out via a phone call. In practice, post-pandemic, these meetings would likely take place at an agreed location on a face-to-face basis. This would increase the cost of introductory meetings due to travel time and travel expenditure. |
| **Expenditure on equipment** | **Assumed total purchase cost** | | **Total annualised equivalent expenditure over project period** | | We have included expenditures on equipment to support the role of the volunteer coordinator. These are based on current estimated purchase prices. Equipment costs are annualised assuming a 3- year life span for the equipment and a 3% discount rate: this enables these types of capital spend to be allocated to the project period in question and recognising that the equipment may continue to be used beyond this point. In practice required expenditure of these type of items when setting up similar programmes in other localities may be higher. | |
| *1x Laptop* | £775 | | £458 | |  |  |
| *1X mobile phone* | £300 | | £177 | |  |  |
| **Total cost per Health Champion** | £337 | | Total cost of staff time plus total equipment expenditure divided by number of active health champions in the feasibility trial (n=25) | | | |
| **Total cost per trial participant** | £ 312 | | Total cost of staff time plus total equipment expenditure divided by number of participants who were randomised in the feasibility trial (n=27) | | | |
