# Supplementary material for: Evaluating a volunteer ‘Health Champions’ intervention supporting people with severe mental illness to manage their physical health: feasibility hybrid randomised controlled trial
Source: BJPsych Open. 2024 Oct 4;10(5):e172. doi: 10.1192/bjo.2024.746 (PMC11536213; doi:10.1192/bjo.2024.746)
Supplement: Williams et al. supplementary material 3 — Williams et al. supplementary material [file S2056472424007464sup003.docx]

HEALTH CHAMPION

JOURNAL

[
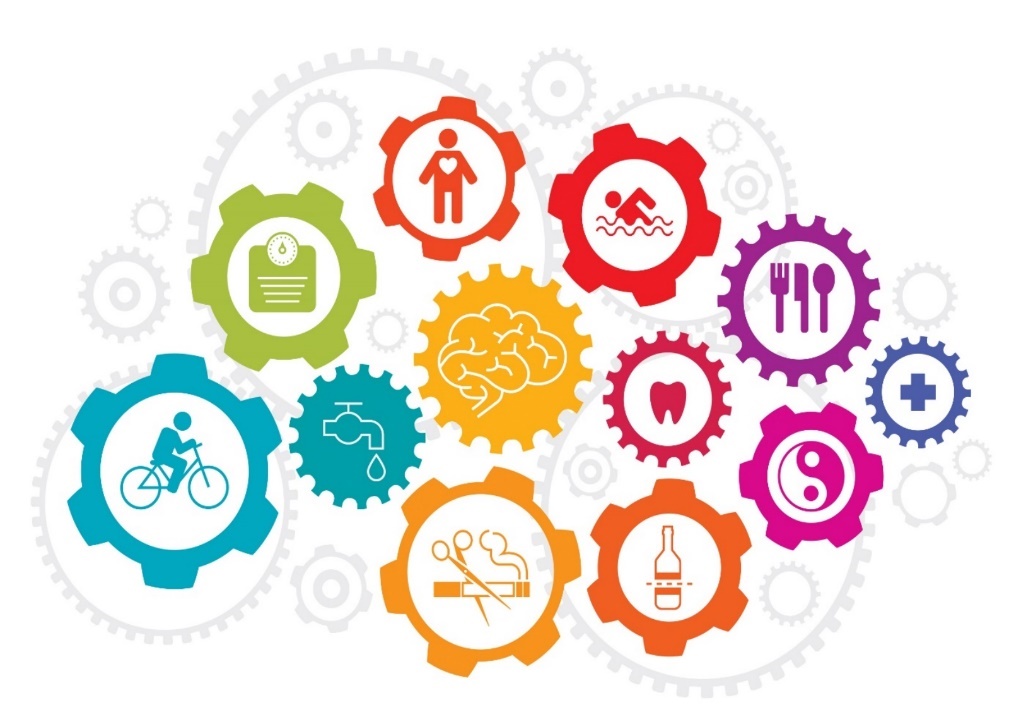
](https://www.google.com/url?sa=i&rct=j&q=&esrc=s&source=images&cd=&cad=rja&uact=8&ved=2ahUKEwiD0Omf3p3iAhULGhQKHfvuCNsQjRx6BAgBEAU&url=https://www.bhru.iph.cam.ac.uk/what-are-the-ways-we-can-change-the-physical-environments-around-us-to-change-behaviour/&psig=AOvVaw2ByYYdx0pL32XFN7lSPVJ4&ust=1558016388969801)[
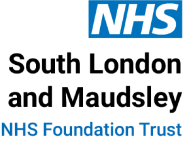
](https://www.google.com/url?sa=i&rct=j&q=&esrc=s&source=images&cd=&cad=rja&uact=8&ved=2ahUKEwiE4tjb553iAhVJ8OAKHarACsoQjRx6BAgBEAU&url=https://www.lewishamdementiasupporthub.org.uk/about/&psig=AOvVaw2AZlIN1XvGIyHAKQxzeasv&ust=1558019080694966)[
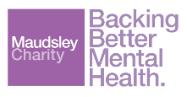
](https://www.google.com/url?sa=i&rct=j&q=&esrc=s&source=images&cd=&cad=rja&uact=8&ved=2ahUKEwj01dzE6J3iAhXHAGMBHURGDaoQjRx6BAgBEAU&url=https://uk.virginmoneygiving.com/charities/maudsleycharity&psig=AOvVaw3TcOoK66ooiiQ0na-eTMWt&ust=1558019352675384)[
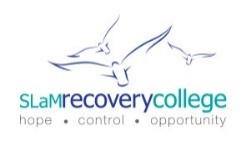
](https://www.google.com/url?sa=i&rct=j&q=&esrc=s&source=images&cd=&cad=rja&uact=8&ved=2ahUKEwjllpiZ6p3iAhUi2-AKHX1hBhAQjRx6BAgBEAU&url=https://www.morleycollege.ac.uk/connect&psig=AOvVaw1oIlkvMqEqsueA_Morz8rY&ust=1558019784898899)[
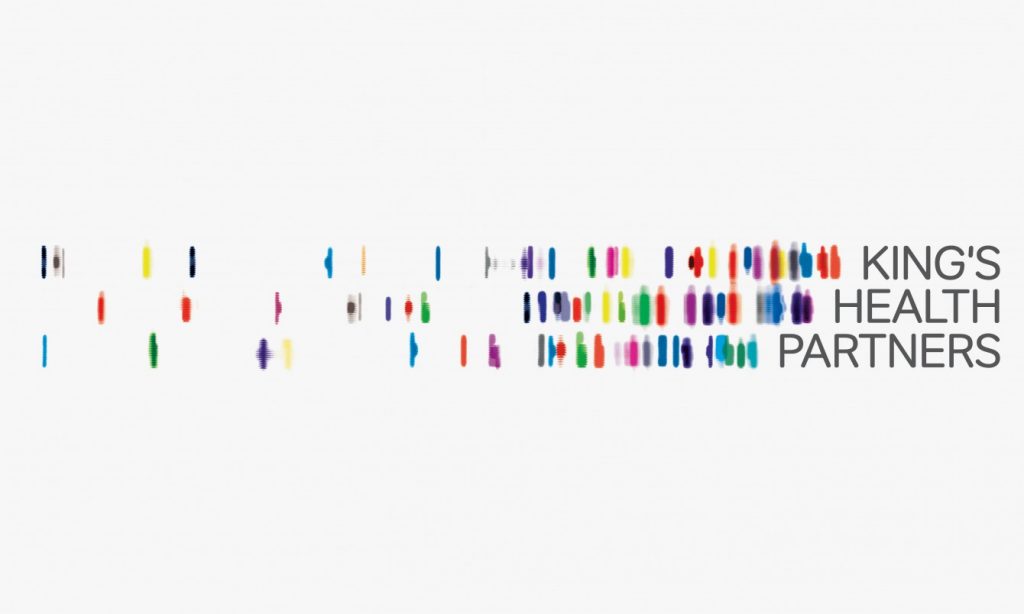
](http://www.google.com/url?sa=i&rct=j&q=&esrc=s&source=images&cd=&cad=rja&uact=8&ved=2ahUKEwjRyLfa7p3iAhUC7eAKHe-JA9IQjRx6BAgBEAU&url=http://www.guysandstthomasbrc.nihr.ac.uk/2016/05/10/brc-director-addresses-kings-health-partners-annual-conference/khp-logo-long/&psig=AOvVaw22XKx6vSdRmJ4I_CDw90BO&ust=1558020964612714)

# **Contents**

[Contents 2](#_Toc10024399)

[Welcome 3](#_Toc10024400)

[Help & Support 4](#_Toc10024401)

[Group Supervision 4](#_Toc10024402)

[Contacts 5](#_Toc10024403)

[How to set SMART Goals 6](#_Toc10024404)

[Record of Meetings 8](#_Toc10024405)

[1^st^ Meeting 8](#_Toc10024406)

[2^nd^ Meeting 8](#_Toc10024407)

This journal belongs to:______________________________

# **Welcome**

Thank you for volunteering to be a Health Champion! We hope that by giving people support to set and achieve personal physical health goal/s, they will be able to maintain and/or improve their physical health. You are key to that process.

Please keep this journal safe.

We would like you to use the journal to keep a record of your meetings with your ‘buddy’. Hopefully, the journal will help you to plan your time, to keep track of any actions that you agree, and to evaluate progress. At the end of the project we will ask to see your journal, to help us understand what may have helped people to achieve their goal/s and what may have been the barriers.

Participants have also been given a journal, which they might ask you to help them complete. Their journal has very similar content. However, the participant journal is theirs’ to keep, they do not have to show us if they do not want to.

# **Help & Support**

We want you to feel supported in your role as Health Champions.

Equally, we are aware that thing’s don’t always go as planned. For example, you may not be able to contact your ‘buddy’, or you might have an emergency that prevents you from attending a meeting.

If you are experiencing any difficulties in fulfilling your role, or even if you just want some brief advice, please do not hesitate to get in contact with any of the programme support team. We will endeavour to help resolve the issue, or at least find an alternative solution. Key contacts are printed on the following page.

## **Group Supervision**

In addition to individual support, throughout the duration of the programme we will facilitate regular group supervision sessions for Health Champions. We will try to make them as accessible as possible, so that you are able to attend. However, they are not compulsory.

The sessions are intended to provide a safe and friendly space to discuss your experience of the Health Champion programme. We will notify you well in advance of when the sessions will be held.

# **Contacts**

| **Name** | **Role / Organisation** | **Telephone** | **Email** |
| --- | --- | --- | --- |
| *Ubong Akpan* | *Volunteer Coordinator-SLaM* |  | *ubong.akpan@slam.nhs.uk* |
| *Isobel Mdudu* | *Volunteer Services Manager-SLaM* |  | *isobel.mdudu@slam.nhs.uk* |
| *Karen Ang* | *IMPHS Project Lead-SLaM* |  | *Karen.ang@slam.nhs.uk* |
| *Ray*  *McGrath* | *IMPHS Lead Nurse-SLaM* |  | *raymond.mcgrath@slam.nhs.uk* |
| *Julie*  *Williams* | *Principal investigator-King’s College London* | *07920 831414* | *julie.williams@kcl.ac.uk* |
|  |  |  |  |
|  |  |  |  |
|  |  |  |  |
|  |  |  |  |
|  |  |  |  |
|  |  |  |  |
|  |  |  |  |

# **How to set SMART Goals**

We have chosen SMART (**S**pecific, **M**easurable, **A**chievable, **R**ealistic, **T**ime-bound) goal setting as an appropriate model to help participants plan their physical health goal/s.

As part of your induction to Health Champions, you will be given brief training on how to support your ‘buddy’ to identify what is important to them about their physical health, and how to help them plot their SMART goal/s.

It is important that you make every effort to help your ‘buddy’ set their goal/s in your first two meetings together. So that you both have a good idea of what you will do, and to make the most of your time together in future meets.

The example on the next page should act as a prompt you to be able to support you buddy complete their SMART goal/s if they need help. But, remember, the goal/s are your buddy’s not yours.

**Example SMART Goal Entry**

| **SMART** | ***EXAMPLE ENTRY 1*** | ***EXAMPLE ENTRY 2*** |
| --- | --- | --- |
| **S**pecific - What is the specific thing you want to be able to do at the end of your time together? | I want to be able to play football with my kids without feeling out of breath. | I want to manage my diabetes better. |
| **M**easurable – How could you measure your progress towards that goal? | Count how many minutes of a game can I play before I feel tired/ out of breath. | Count how many times my blood sugar is in the ‘good’ range or how many times I feel unwell/have bad days because of my diabetes. |
| **A**chievable - Is the goal feasible? Do you have the right equipment and skills? | I am able to play football and I feel well enough to try. | I have the equipment to monitor my blood sugar and I am registered with a GP. |
| **R**ealistic - Are sufficient resources available? | My health champ will help me find a space to practice or exercise. | My health champ will help me get to my regular health checks and local support for people with diabetes and find meal plans. |
| **T**ime-Bound – How long will it take to achieve your goal or how far do you expect to be in 1/2/3/4 month(s)? | My current level physical of activity is low so I will need to start slowly and build up exercises over 3 months. | My diabetes is managed ok but I have some health check to catch up on so at least 4 months maybe more. |

# **Record of Meetings**

| 1^st^ Meeting Date: | |
| --- | --- |
| Did the meeting go well? | |
| Did the participant set a SMART goal?  Yes/ NO | If Yes, what was the participant’s goal/s? |
| Were there any challenges or issues arising? | |
| Actions (is there anything to do before the next meeting?) | |
| When & Where is the next meeting? | |
| 2^nd^ Meeting Date: | |
| Did the meeting go well? | |
| Has the participant agreed a SMART goal?  YES / NO | If YES, what is the participant’s goal/s? |
| Were there any challenges or issues arising? | |
| Actions (is there anything to do before the next meeting?) | |
| When & Where is the next meeting? | |

| Meeting Date: | |
| --- | --- |
| Did the meeting go well? | |
| Has progress been made in achieving the set goal/s?  Yes/ NO | If Yes, what was the progress? |
| Were there any challenges or issues arising? | |
| Actions (is there anything to do before the next meeting?) | |
| When & Where is the next meeting? | |

| Meeting Date: | |
| --- | --- |
| Did the meeting go well? | |
| Has progress been made in achieving the set goal/s?  Yes/ NO | If Yes, what was the progress? |
| Were there any challenges or issues arising? | |
| Actions (is there anything to do before the next meeting?) | |
| When & Where is the next meeting? | |

| Meeting Date: | |
| --- | --- |
| Did the meeting go well? | |
| Has progress been made in achieving the set goal/s?  Yes/ NO | If Yes, what was the progress? |
| Were there any challenges or issues arising? | |
| Actions (is there anything to do before the next meeting?) | |
| When & Where is the next meeting? | |

# **Notes**

(A space for you to jot down anything you may find useful or that you want to remember)
