# Supplementary material for: Evaluating a volunteer ‘Health Champions’ intervention supporting people with severe mental illness to manage their physical health: feasibility hybrid randomised controlled trial
Source: BJPsych Open. 2024 Oct 4;10(5):e172. doi: 10.1192/bjo.2024.746 (PMC11536213; doi:10.1192/bjo.2024.746)
Supplement: Williams et al. supplementary material 4 — Williams et al. supplementary material [file S2056472424007464sup004.docx]

Health Champions Follow up questions

Health Champions

We would like to ask you some questions about your experience of being a Health Champion in this study. This will help us to understand more about this role and whether we need to make any changes.

1. Please tell us about your experience of taking part in the study? (prompts-what did you enjoy? What did you find useful? What did you find challenging? What did you like/not like about being a Health Champion?
2. How did you find our communication with you throughout? (prompts: what did you find was good? What did you find was not so good?)
3. What could we improve in our communication and support for Health Champions?
4. How did you find the recruitment process?
5. How did you find the matching process?
6. To what extent did you feel that you had enough information to become a Health Champion?
7. How useful was the welcome session in part 1 ‘Becoming a health champion’?
8. How useful was the welcome session part 2 ‘Beginning the conversation’?
9. Did you attend any of the group supervision sessions? if yes, did you find them helpful? how were they helpful? if you didn’t attend, did you have regular contact to have supervision/support?
10. At the beginning of the study you said that you wanted to get - Seeing improvements-making a bond which is rewarding for both. To what extent did this happen? How did this change during the time you were taking part?
11. At the beginning of the study you said that you thought Communication. Boundaries.
12. would be a challenge. To what extent was this the case?
13. What other challenges were there that you did not anticipate?
14. What goals did the person you support set?
15. How did you help them to write down/keep track of their goals?
16. How did you assess whether these goals had been achieved?
17. How long did it take to set these goals with the person? (re: relationship building?)
18. How did they work towards these goals? Tell me more about that and how you supported this?
19. What were the barriers to them setting and working towards their goal(s)? What other barriers do you think they had to taking part? (Barriers)
20. What factors helped them to set and work towards their goal(s)? (facilitators). What other factors do you think helped them to take part in the study?
21. How do you think they found the intervention/programme useful?
22. How did you use the journal? To what extent was it helpful?
23. How did you get on with the person you supported?
24. How often did you have contact with them?
25. How did you communicate with each other? (how often, what methods did you use?)
26. Do you think as this contact was about right/too much/too little? Please explain why?
27. To what extent did you find that you had the time to be a Health Champion? How did being a Health Champion fit in with your everyday life activities? (Feasibility)
28. What else did you discuss with the person you supported? How did you focus on supporting their physical health? What other things did you support them with?(Fidelity)
29. To what extent were there any things arising that you were not expecting to support them with? (unintended consequences)
30. Do you think that 9 months to support the person was long enough/about right/too short? Please explain why?
31. What do you think the person got out of having a Health Champion?
32. What contact did you have with their care team? If you had contact, how did you find this?
33. How did you find the ending was with the person? How easy/difficult, any issues?
34. What did you feel that you learned from being a Health Champion?
35. What benefits did you get out of being a health Champion?
36. What other volunteering possibilities would you be interested in?
37. Would you like us to provide you with a summary of the findings of this study?
38. Would you be interested in helping us to publicise the findings? (thinking about where to publicise, being interviewed etc)
39. Is there anything else you would like to tell us?
