# Supplementary material for: Evaluating a volunteer ‘Health Champions’ intervention supporting people with severe mental illness to manage their physical health: feasibility hybrid randomised controlled trial
Source: BJPsych Open. 2024 Oct 4;10(5):e172. doi: 10.1192/bjo.2024.746 (PMC11536213; doi:10.1192/bjo.2024.746)
Supplement: Williams et al. supplementary material 5 — Williams et al. supplementary material [file S2056472424007464sup005.docx]

Health Champions Follow Up Questions-

Participant questions

We would like to ask you some questions about your experience of the Health Champions study and working with your Health Champion. This will help us to understand more about the programme and to know if we need to make any changes.

1. Please tell me about taking part in the study-what was your experience like? (prompts-did you enjoy it? Did you find it useful? What did you like/not like about taking part?)
2. How did you find the communication from the research team when you started? (prompts-did we give you enough information on what was involved? Were the written documents (PIS and consent form) easy to understand? Did you feel that you could contact us if you had any questions and did you know how to contact us? Is there anything we could have changed or done better?)
3. How did you find the process of matching you with a Health Champion? (was it difficult? Easy? Please explain)
4. How did you find the time that it took us to match you with a Health Champion?
5. At the beginning of the study we asked you what you wanted to get out of taking part-can you remember what you said? (if not, prompt -this is what you said that you wanted to get out of taking part)
6. Was that what you worked on with your health champion, or did this change? If it changed, please explain why?
7. How did you meet your goals?
8. To what extent did you find that having a Health Champions was a good fit for you? Did it work for you? (Feasibility)
9. How relevant or useful was having a Health Champion to meet your needs? (Appropriateness)
10. How did having support from your Health Champion help you to set and work towards your physical health goals? (prompts-what were your goal(s)? To what extent did you meet your goals?)
11. What was it about working with your Health Champion that helped you with this? What didn’t help with this? (facilitators)
12. What barriers or challenges did you have to setting and working towards your goal(s)? what other barriers did you have to working with your Health Champion? (Barriers)
13. How did you get on with your Health Champion?
14. How often did you have contact with your Health Champion?
15. Would you say this amount of contact was about right/too much/too little? Please explain why?
16. What did you discuss with your Health Champion? How did you focus on managing your physical health? (Fidelity)
17. How did the Health Champion help you with anything else besides supporting you to manage your physical health? If yes, please explain what they helped you with? (unintended consequences)
18. To what extent were there any things arising that you were not expecting when working with your Health Champion? If yes, was this positive/negative/neither? (unintended consequences)
19. We gave you a journal when you started. How did you feel about this journal? (prompts: did you use it? If so, was it helpful? what did you use it for? would you consider sharing it?, did it help you keep track of your progress? Or did you use other ways of doing this-eg an app, diary)
20. Do you think 9 months with the health champion was long enough/too long/about right? Can you tell us why?
21. How did you feel when your time with your Health Champion ended?
22. To what extent do you feel that you will be able to maintain any positive changes you have made in your everyday life?
23. How will you continue to work towards looking after your physical health?
24. What will you be able to do now that you were not able to do before taking part?
25. What things won’t you be able to do now that you are no longer part of the study?
26. What alternative support to help you with physical health will you look for?
27. Would you recommend health champion support to a friend? If yes, why. If not, why not?
28. Was your care coordinator aware that you had a Health Champion? If yes, did they ask you about it?
29. Would you like us to send you a summary of the findings from the study? (lay summary and academic paper)?
30. Would you be interested in helping us to disseminate the findings? (eg helping to think about where to publicise the findings)
31. Is there anything else you would like to tell us about your experience of having a Health Champion?
